# External validation of the CREST model to predict early circulatory-etiology death after out-of-hospital cardiac arrest without initial ST-segment elevation myocardial infarction

**DOI:** 10.1186/s12872-023-03334-4

**Published:** 2023-06-20

**Authors:** Zana Haxhija, David B Seder, Teresa L May, Christian Hassager, Hans Friberg, Gisela Lilja, Ameldina Ceric, Niklas Nielsen, Josef Dankiewicz

**Affiliations:** 1 Department of Clinical Sciences, Anesthesia and Intensive Care, Lund University, Skane University Hospital, Malmo, Sweden; 2 Department of Critical Care Services, Maine Medical Center, Portland Maine, USA; 3 Department of Cardiology, Rigshospitalet, University of Copenhagen, Copenhagen, Denmark; 4 Department of Clinical sciences, Neurology, Lund University, Skane University Hospital, Lund, Sweden; 5 Department of Clinical Sciences, Anesthesia and Intensive Care, Lund University, Helsingborg Hospital, Helsingborg, Sweden; 6 Department of Clinical Sciences, Cardiology, Lund University, Skane University Hospital, Lund, Sweden; 7 Division of Anesthesia and Intensive Care, Department of Clinical sciences Lund, Lund University, Skane University Hospital, Carl Bertil Laurells gata 9, Malmo, 205 02 Sweden

**Keywords:** Cardiac arrest, Resuscitation, Prediction model

## Abstract

**Background:**

The CREST model is a prediction model, quantitating the risk of circulatory-etiology death (CED) after cardiac arrest based on variables available at hospital admission, and intend to guide the triage of comatose patients without ST-segment-elevation myocardial infarction after successful cardiopulmonary resuscitation. This study assessed performance of the CREST model in the Target Temperature Management (TTM) trial cohort.

**Methods:**

We retrospectively analyzed data from resuscitated out-of-hospital cardiac arrest (OHCA) patients in the TTM-trial. Demographics, clinical characteristics, and CREST variables (history of coronary artery disease, initial heart rhythm, initial ejection fraction, shock at admission and ischemic time > 25 min) were assessed in univariate and multivariable analysis. The primary outcome was CED. The discriminatory power of the logistic regression model was assessed using the C-statistic and goodness of fit was tested according to Hosmer-Lemeshow.

**Results:**

Among 329 patients eligible for final analysis, 71 (22%) had CED. History of ischemic heart disease, previous arrhythmia, older age, initial non-shockable rhythm, shock at admission, ischemic time > 25 min and severe left ventricular dysfunction were variables associated with CED in univariate analysis. CREST variables were entered into a logistic regression model and the area under the curve for the model was 0.73 with adequate calibration according to Hosmer-Lemeshow test (p = 0.602).

**Conclusions:**

The CREST model had good validity and a discrimination capability for predicting circulatory-etiology death after resuscitation from cardiac arrest without ST-segment elevation myocardial infarction. Application of this model could help to triage high-risk patients for transfer to specialized cardiac centers.

## Introduction

Out-of-hospital cardiac arrest (OHCA) is a leading cause of death in Europe and the United States. Only one in four patients achieve return of spontaneous circulation (ROSC) and the overall survival to hospital discharge for patients admitted to the intensive care unit after successful resuscitation is approximately 40% [1–3], though there are considerable regional and intra-center variations in outcome [4–6].

Two-thirds of subsequent deaths occur from hypoxic-ischemic brain injury [7, 8], and about one-third from circulatory-etiology death (CED), which includes recurrent cardiopulmonary arrest, progressive refractory shock and multiorgan system failure. CED accounts for most deaths in the first three days [9, 10]. Raw or processed electroencephalography and other modalities allow for very early assessment of brain injury-severity after resuscitation, and potentially for neurological risk stratification [11–15]. It would be useful to know the competing risk of CED when triaging cardiac arrest patients. However, there is no established prediction tool in the triage of patients to interventions based on the risk of CED [16]. In cases of ST-segment-elevation myocardial infarction (STEMI) on initial ECG after resuscitation, American and European guidelines recommend urgent percutaneous angiography [17, 18]. The majority of post-resuscitation intensive care unit admissions, however, are without STEMI and the ideal treatment pathway for these patients remains unclear [19–22].

The CREST-model is a risk stratification tool developed to help clinicians decide appropriate pathways for patients without STEMI on initial electrocardiogram (ECG). It was retrospectively derived in a cohort of patients from the International Cardiac Arrest Registry [23]. The model predicts CED based on variables readily available early after resuscitation; known Coronary artery disease, non-shockable initial heart Rhythm, initial Ejection fraction < 30%, Shock at admission, and Time to ROSC more than 25 min, creating a cumulative risk index. In the derivation study, a linear increase in the likelihood of circulatory-etiology death was seen with incremental increases in the CREST score, which ranged from 0 to 5 [23]. The model was internally validated within the same registry, using a random sample of two-thirds of the patients. To date, the CREST-model has been externally validated only in one single-center study, including 211 OHCA patients [24]. The aim of this study was to determine the validity of the CREST model in the Target Temperature Management (TTM) trial cohort [25].

## Methods

### Study population

The TTM-trial was a multinational assessor-blinded trial of unconscious (GCS < 8), adult (age > 18 years) cardiac arrest patients with ROSC after OHCA of a presumed cardiac cause, randomized to temperature management at either 33^o^C or 36^o^C [25]. It was performed in 36 participating centers worldwide and included standardized data definitions with detailed descriptions of the hospital course and patient outcomes. Ethical committees in each participating country approved the TTM-trial protocol and informed consent was waived or obtained according to national legislations, in line with the Helsinki declaration. The trial randomized 950 patients resuscitated from OHCA from 2010 to 2013 who remained unconscious after ROSC. In total, 939 patients were included in the modified intention to treat analysis. The main exclusion criteria were ischemic time to screening > 4 h, suspected or confirmed intracranial bleeding, suspected or confirmed acute stroke and unwitnessed asystole as the initial rhythm [25]. Patients in both groups were sedated, endotracheally intubated, and mechanically ventilated. The intervention period lasted for 36 h and was followed by protocolled prognostication.

This sub-study was conducted using data from the TTM-trial. Patients with STEMI on their initial post resuscitation ECG were excluded from the study and patients with missing data relevant to analysis were excluded from the logistic regression analysis. The majority of excluded patients with missing data were those that did not undergo an initial echocardiogram.

### Data collection, CREST variables and outcomes

Data were collected from an electronic case report form on the TTM-server; resuscitation data and outcomes are consistent with the Utstein-style for reporting cardiac arrest and patient characteristics. The CREST variables of interest were as described above [23]. Ischemic time or time to ROSC was defined as time from cardiac arrest to ROSC for witnessed arrest and as time from emergency call to ROSC for unwitnessed arrest. Shock at admission was defined as a systolic blood pressure of < 90 mmHg for > 30 min, or the need for supportive measures (fluid loading, vasopressors, inotropic medication and/or intra-aortic balloon pump) to maintain a systolic blood pressure of > 90 mmHg and/or end-organ hypoperfusion (cool extremities, urine output of < 30ml/h). Blood pressure was determined by invasive blood pressure monitoring with arterial catheter. The initial echocardiogram was obtained on admission or on the first day in the ICU. The primary outcome of interest was circulatory-etiology death determined by the treating physician.

### Statistics

Comparisons between the groups according to outcome were made on baseline characteristics and clinical characteristics with unadjusted analysis to assess for associations. Categorical data were analyzed using Chi-square tests and are displayed as counts and percentages. Continuous variables were analyzed using Students t-test or Wilcoxon rank sum test and are presented as mean values +/- standard deviation (SD) or as median values with interquartile range (IQR). Baseline and clinical characteristics for patients with missing data relevant to analysis were compared to the study cohort in univariate analysis.

Multivariable logistic regression analysis was used to assess the independent association between CREST model variables and outcome with CED as the dependent variable, creating an un-weighted model. Results from the regression model are reported as odds ratios (OR) with a 95% confidence interval (CI). Goodness of fit for the logistic regression model was assessed with the Hosmer-Lemeshow test, creating 8 groups, and a *p* value of > 0.05 was considered to represent an adequate model fit. The discriminatory power of the logistic regression model was assessed with area under the receiver operating characteristic curve (ROC). Predicted and observed incidence of CED were compared according to the CREST-score. All statistical analyses were performed using SPSS software version 25.0 and a *p*-value of < 0.05 was considered to be significant.

## Results

### Patient characteristics and unadjusted analysis according to CED

**Fig. 1:**
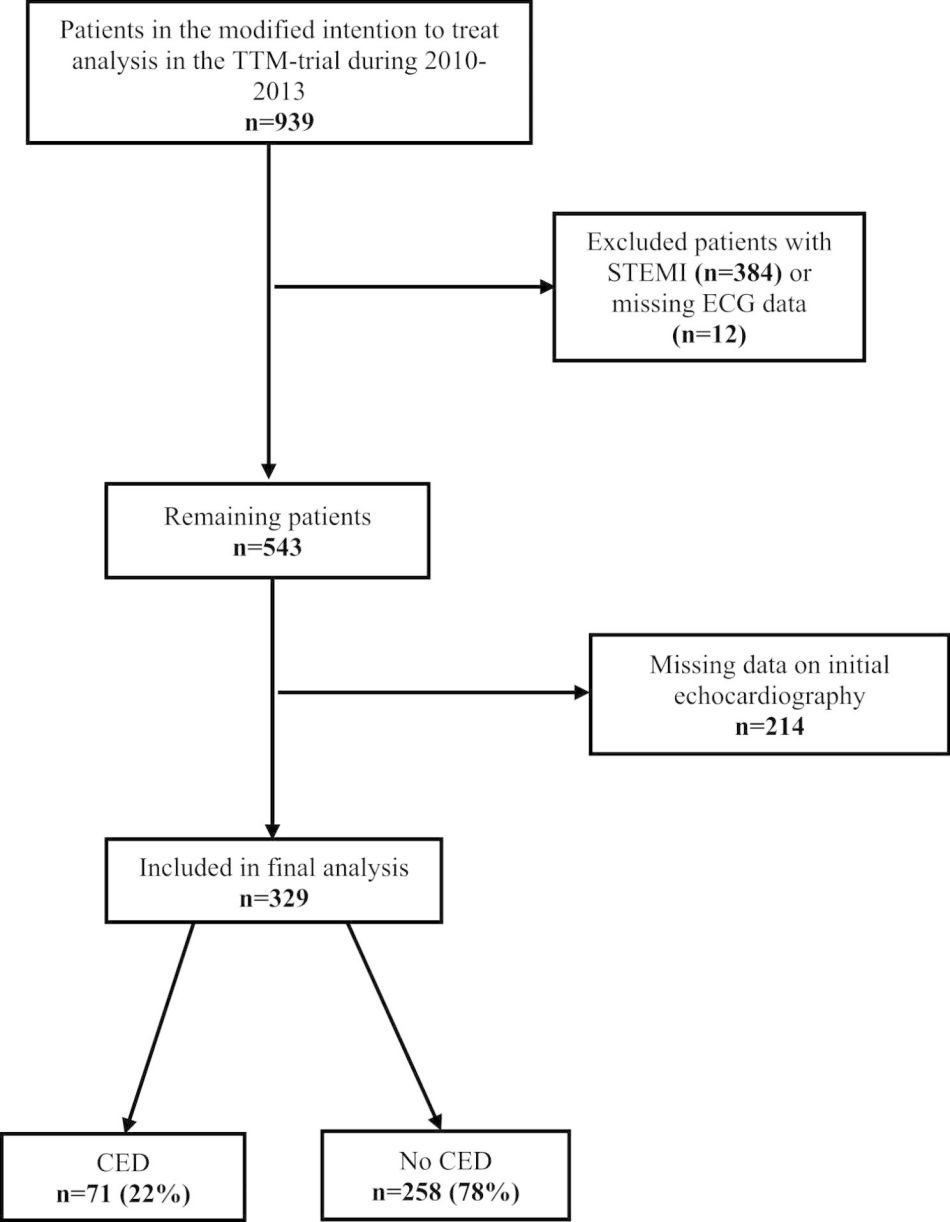
Flow diagram for patient selection

Among the 939 OHCA patients that were entered into the TTM-trial database between 2010 and 2013, we excluded patients who met the criteria for STEMI (n = 384) or had missing ECG data (n = 12). Primary univariate analyses were made on the remaining 543 patients. Additionally, 214 patients were excluded because of missing data on admission echocardiogram, leaving 329 patients for final analysis. Of these, 71 met the criteria for circulatory-etiology death and 99 patients died from other causes including neurological-etiology death. 159 patients survived to the end of the trial (Fig. 1).

Patients in the modified intention to treat analysis in the Target Temperature Management (TTM)-trial during 2010–2013. CED indicates circulatory-etiology death; no CED includes patients who survived or died from other causes including neurological-etiology death. 329 patients were included in final analysis.

Table 1 summarizes the univariate associations of demographics and clinical characteristics with outcomes. Older age (*p* = < 0.001), previous myocardial infarction (*p* = 0.012), previous arrhythmia (*p* = 0.001), history of ischemic heart disease (*p* = < 0.001), non-shockable initial heart rhythm (*p* = 0.011), longer ischemic time (*p* = 0.004), shock at admission (*p* < 0.001), coronary angiography at any time (*p* = 0.001) and initial left ventricular ejection fraction < 30% (*p* = 0.008) were variables associated with CED. No differences in the two groups of outcomes were observed regarding urgent angiography (*p* = 0.117) and percutaneous coronary intervention (PCI) at any time (*p* = 0.831).

Univariate analysis of baseline and clinical characteristics of the study cohort compared to the group of patients with missing echocardiogram found no significant difference between the groups.

**Table 1:** Univariate analysis of associations between demographics and clinical characteristics with outcomes, in patients included in the primary analysis

Variable	CED, n (%)	No CED, n (%)	*p* value
n	106	437	
Age in years, mean (+/-SD)	72 (10)	63 (13)	< 0.0001
Gender, female	27 (25)	86 (20)	0.236
Hypertension	54 (51)	173 (40)	0.088
Heart failure	16 (15)	31 (7)	0.015
Previous myocardial infarction	36 (34)	95 (22)	0.012
Previous arrhythmia	37 (35)	86 (20)	0.001
Diabetes	23 (22)	67 (16)	0.150
Ischemic heart disease	55 (52)	124 (28)	< 0.001
Witnessed arrest	92 (87)	396 (91)	0.321
Bystander CPR	67 (63)	316 (72)	0.084
Non-shockable initial heart rhythm	37 (35)	98 (22)	0.011
Ischemic time in min, median (IQR)	31 (19–45)	25 (16–37)	0.004
Ischemic time > 25 min	63 (59)	194 (44)	0.007
Shock at admission	38 (36)	37 (9)	< 0.001
Urgent angiography	44 (42)	221 (51)	0.117
Any angiography	52 (49)	294 (67)	0.001
Any PCI	25 (24)	110 (25)	0.831
Initial left ventricular ejection fraction < 30%	28 (39)	59 (23)	0.005

*CED indicates circulatory-etiology death; no CED includes patients who either survived or died from other causes including neurological-etiology death; IQR, interquartile range; PCI, percutaneous coronary intervention; and CPR, cardiopulmonary resuscitation

† p values were derived using Student´s t-test test for continuous age, Wilcoxon rank sum test for ischemic time in min and Pearson´s chi-squared test for categorial data

### Analysis of the CREST model

**Fig. 2:**
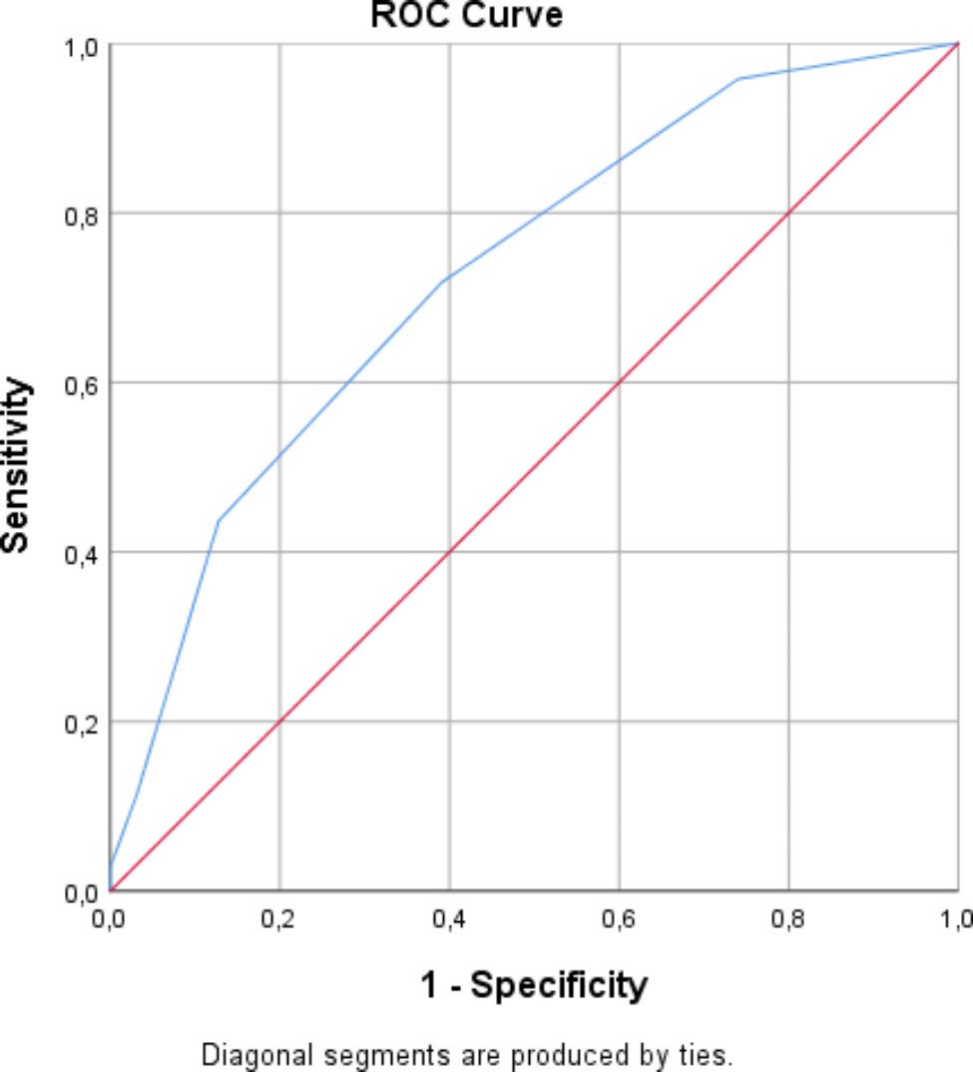
Receiver operating characteristic curve (ROC curve) for the CREST-model The CREST model had a good discrimination capability for predicting circulatory-etiology death, with an area under the curve of 0.73 (confidence interval [CI], 0.66–0.79). The optimal cut-off point was at a CREST score of 2

Table 2 displays the result of the multivariable logistic regression model including the CREST variables with CED as the dependent variable: coronary artery disease (OR, 2.52; p = 0.003), initial non-shockable rhythm (OR, 2.39; p = 0.006), initial left ventricular ejection fraction < 30% (OR, 1.60; p = 0.151), shock at admission (OR, 6.13; p = 0,001) and ischemic time > 25 min (OR, 1.32; p = 0,36). The Hosmer-Lemeshow test showed an adequate goodness of fit for the CREST-model, as evidenced by a non-significant *p* value of 0.602. The model had a good discrimination capability with an area under the curve of 0.73 (CI, 0.66–0.79), as presented in Fig. 2.

**Table 2:** Multivariable logistic regression model of CREST variables with circulatory-etiology death as the dependent variable

Variable	Weight	OR	95% CI	p-value
History of coronary artery disease	1	2.52	1.37–4.62	0.003
Non-shockable initial heart rhythm	1	2.39	1.29–4.43	0.006
Initial left ventricular ejection fraction < 30%	1	1.60	0.84–3.05	0.151
Shock at admission	1	6.13	3.04–12.33	< 0.001
Ischemic time > 25 min	1	1.32	0.73–2.37	0.356

* CI indicates confidence interval and OR indicates odds ratio

**Fig. 3:**
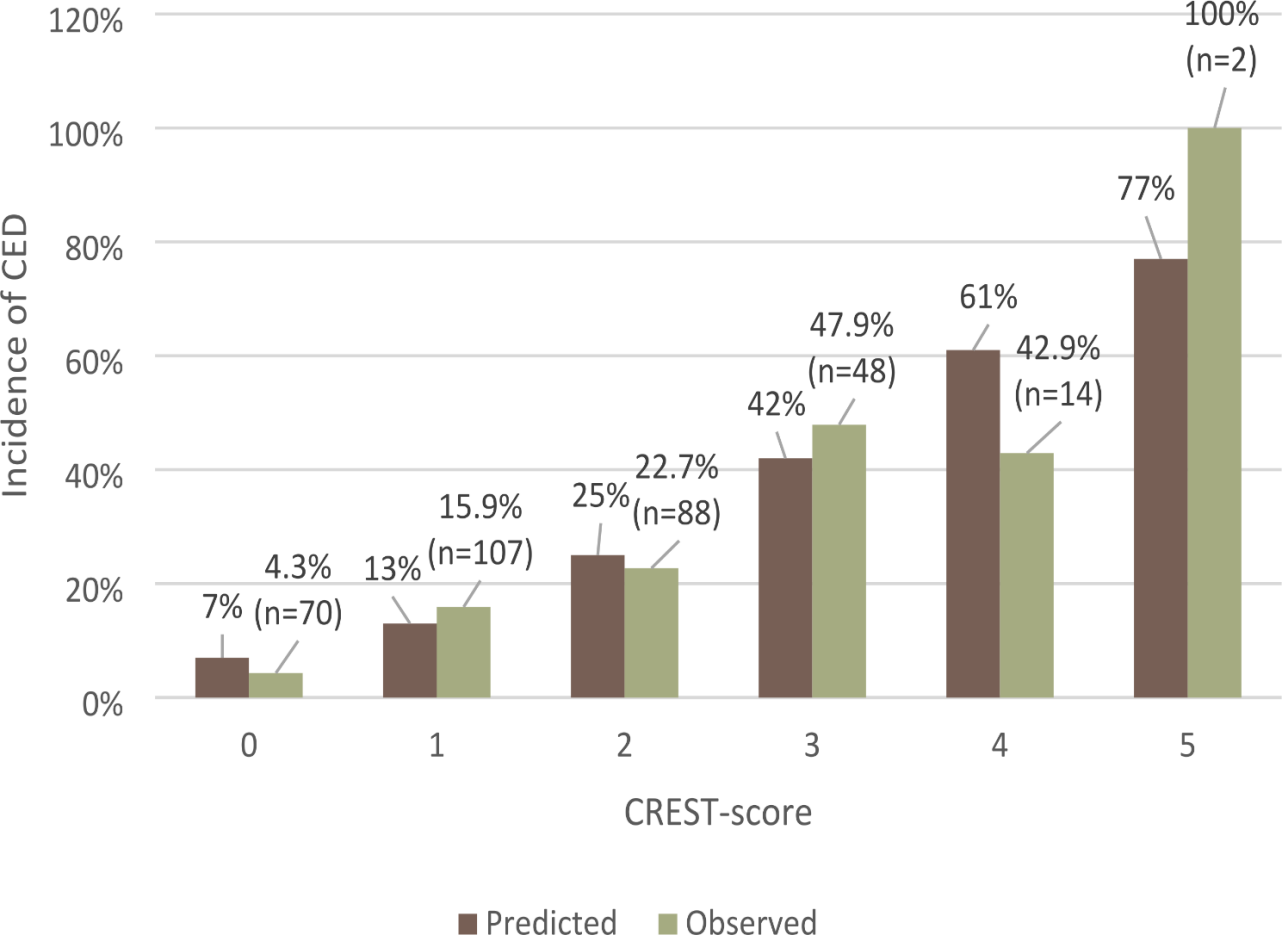
Observed versus predicted incidence of CED according to incremental increases of CREST-variables (CREST score)

Figure 3 shows an increase in the predicted and observed incidence for CED with incremental increases in number of CREST-variables. 329 patients had a CREST-score ranging from 0 to 5, with a score of 4–5 being the least common patient group.

A linear increase in likelihood of circulatory-etiology death (CED) is seen with incremental increases in CREST-variables constituting the CREST score: history of coronary artery disease, non-shockable initial heart rhythm, initial left ventricular ejection fraction < 30%, shock at admission, and ischemic time more than 25 min. *n* = total number of patients in each observed group according to CREST-score.

## Discussion

In this validation study, application of the CREST variables to the TTM-trial dataset generated a ROC curve with an area under the ROC curve of 0.73, confirming the model´s ability to identify patients at risk of circulatory-etiology death at the time of hospital admission with good precision. These results are similar to the two previous published validations on the CREST-model, generating ROC-curves of 0.68 and 0.88 respectively [23, 24]. Observed results in each CREST-category closely approximated the prior study, suggesting that while there may be a range of risk in each category, the incremental increase in risk of circulatory-etiology death with an increasing score may be robust enough to use for clinical triage and individualized decision-making.

Prior studies of post-resuscitation care have mainly focused neurological outcomes after cardiac arrest since this is the most common mode of death for these patients [8]. However, circulatory-etiology death, including multiorgan failure and progressive shock, remains the driver for about one third of deaths. This underscores the importance of identifying high-risk patients that might benefit from triage to specialized cardiac centers since possible interventions for these patients may include early revascularization, mechanical cardiac support, or increased hemodynamic monitoring.

Although TTM is a standard of care for many OHCA patients, guidelines do not make explicit recommendations for patients presenting with shock at admission [26]. Prior studies have indicated that hypothermia improves hemodynamic parameters and may reduce mortality in patients presenting with cardiogenic shock [27–29], although there are conflicting results. The TTM-trial found no differences in mortality or poor neurologic outcome between targeting a core temperature of 33^o^C or 36^o^C in survivors of cardiac arrest; in the subgroup of patients presenting with moderate shock, results did not differ significantly either [25]. These findings are supported by other recent sub analyses of the TTM-trial, creating further uncertainty with the potential benefit of hypothermia in patients with shock [30–32]. One could hypothesize that subgroups of patients with different degrees of neurological injury and circulatory impairment might respond differently to therapy and could benefit from individualized treatment regimens. The potential of matching post-resuscitation care with the type and severity of injury after cardiac arrest is further supported by a study of patients resuscitated from cardiac arrest identifying one cardiac risk group and one neurological dysfunction group. About two thirds of patients determined to have a mild brain injury did not receive adequate circulatory support and one in five of these patients met the end point criteria for CED [33]. Some patients with a more severe brain injury, as determined by processed electroencephalography, received urgent revascularization but later died from neurologic death [33]. These results underscore the fact that the availability of an early risk assessment tool could be a key in the triage of patients to treatment pathways after resuscitation from cardiac arrest, although it should be recognized that there exists substantial overlap; many patients with coronary occlusion and cardiogenic shock also have substantial, and un-survivable brain injury.

Our validation of the CREST model was made on data collected from a large number of both European and Australian sites, which might extend the generalizability; however, the different treatment protocols used are difficult to fully adjust. The discriminatory power of the CREST model is in a range similar to other major clinical prediction tools regarding cardiovascular risk assessment [34, 35]. For risk equations to be useful in clinical practice, they should also be well calibrated so that predicted risks are similar to observed disease incidence. In this study, in addition to demonstrating good discrimination, the observed and predicted incidence of CED were also similar, indicating good calibration for the prediction model. The study, however, has several limitations. Our results apply to patients with moderate shock since an irreversible severe shock state (SBP < 80 mmHg despite all supportive measures) was an exclusion criterion in the TTM-trial. Also, the definition of shock includes both subjective and objective measurements. Cool extremities, that was used in the definition of shock in this study, is a very subjective measure of tissue perfusion. The variables in the CREST-model are given an equal weight, although the variables had different odds ratios. Shock at admission had a higher odds ratio and one could therefore hypothesize that shock at admission would have a higher weight. Because of the lack of an admission echocardiography, about 200 patients were excluded from the study limiting its generalizability of the study or creating selection bias. These missing data indicate the logistic difficulty of recording a detailed echocardiographic assessment (during all hours of the day), although a binary assessment of the ejection fraction as severely reduced or not might be easier to determine. However, further analyses were made for comparisons between the groups with missing versus no missing echocardiogram, and no significant differences were observed between the two groups.

In the present study, the CREST model was validated in an independent set of patients and showed similar results to the previous validation study, with regard to predicting circulatory-etiology death in comatose patients after cardiac arrest. Application of this model could help to triage high-risk patients for transfer to specialized cardiac centers.

## Data Availability

The dataset used and/or analyzed during the current study is available from the corresponding author upon reasonable request.

## Abbreviations

OHCA: Out-of-hospital Cardiac Arrest
ROSC: Return of spontaneous circulation
CED: Circulatory-etiology death
STEMI: ST-segment-elevation myocardial infarction
ECG: Electrocardiogram
SD: Standard deviation
TTM-trial: Target Temperature Management trial
OR: Odds ratio
CI: Confidence interval
ROC: Receiver operating characteristic curve
